# HF-Ultrasonography to Quantify Skin Atrophy in Patients with Inflammatory Rheumatic Diseases Treated with Courses of Glucocorticoids

**DOI:** 10.3390/diagnostics15050619

**Published:** 2025-03-04

**Authors:** Antonia Schuster, Andreas Horn, Florian Günther, Martin Fleck, Wolfgang Hartung, Boris Ehrenstein

**Affiliations:** 1 Department of Nephrology, University Hospital Regensburg, Franz-Josef-Strauß-Allee 11, 93053 Regensburg, Germany; 2 Department of Rheumatology and Clinical Immunology, Asklepios Medical Center Bad Abbach, 93077 Bad Abbach, Germany; 3 Department of Internal Medicine I, University Hospital Regensburg, 93053 Regensburg, Germany; 4 Faculty of Medicine, University of Regensburg, 93053 Regensburg, Germany

**Keywords:** prednisolone, glucocorticoids, ultrasonography, drug-related side effects, skin atrophy

## Abstract

**Background**: Prolonged courses of glucocorticoids (GCs) for patients suffering from inflammatory rheumatic diseases (IRDs) are associated with adverse effects. High-frequency ultrasonography (HFUS) has been utilized to quantify skin changes during short-term topical GC treatment. We aimed to quantify skin atrophy in IRD patients treated systemically with prolonged courses of GCs. **Methods**: We performed a cross-sectional study comparing patients with IRDs and GC treatment who presented with clinically evident skin atrophy to a matched cohort (1:1) without IRDs and GC treatment. Skinfold measurements, utilizing a standardized caliper, and B-mode HFUS images, utilizing an 18 MHz linear sonography probe, were acquired at back-of-hand, cubital, and dorsal midfoot regions and then compared between both groups. **Results**: A total of 53 GC-treated IRD patients (33 (62%) women, mean age 66.4 (±10.0) years, GC treatment median 8.0 (1.0–47.0) years) were compared to 53 subjects without IRDs and GC treatment (32 (60%) women, 65.9 (±11.3) years). Skinfold thickness measured at the back of hands [1.7 (±0.4) vs. 2.1 (±0.5) mm, *p* < 0.001], but not at the cubital [6.7 (±2.7) vs. 7.1 (±3.0) mm] or dorsal midfoot [3.6 (±3.7) vs. 4.1 (±3.4) mm] areas, showed a significant difference between the groups. In comparison, all areas displayed statistically significant different cutaneous thickness in the evaluation by HFUS: hand 0.66 (±0.12) vs. 0.82 (±0.18), *p* < 0.001; cubital 0.86 (±0.15) vs. 1.00 (±0.21), *p* < 0.001; and midfoot 0.76 (±0.16) vs. 0.94 (±0.18), *p* < 0.001. **Conclusions**: This study revealed significantly lower values in the measured cutaneous thickness by HFUS for GC-treated patients with IRDs compared to persons without IRD and GC treatment.

## 1. Introduction

Glucocorticoids (GCs) are the most widely used class of anti-inflammatory drugs in medicine. Side effects of GCs are common and often problematic, ranging from mild cases of acne to severe manifestations of Cushing syndrome [1]. A population-based self-administered survey of US-American patients from 2001 to 2002 receiving systemic long-term GC-treatment reported skin bruising or thinning in 45% up to 65% of patients, with an increasing prevalence stratified by cumulative GC-dose, <1.7 vs. >4.7 g of prednisolone-equivalent, respectively [2]. A cross-sectional study, surveying patients with rheumatoid arthritis from 2012 to 2013 in two European academic centers, demonstrated rates of self-reported skin atrophy in up to 41% of patients receiving long-term GCs compared to 6% of patients never treated with GCs [3].

High-frequency ultrasonography (HFUS) has been an established method for imaging the human skin for several decades [4]. While HFUS utilizing specialized devices with transducer frequencies between 20 and 70 MHz has been employed in a wide variety of dermatological research, clinical utilization in everyday dermatologic practice is often limited to lymph node evaluations in melanoma patients [4] utilizing standard clinical devices with 12 to 15 MHz transducers. In research settings, short-term (2 to 8 weeks) topical GC treatment of skin diseases reduced total skin thickness measured by HFUS up to 25% compared to baseline measurements, but these skin changes were transient and returned to baseline after 2 weeks of GC cessation, if studied [5]. Although HFUS has been utilized to study skin changes in rheumatic patients with systemic sclerosis [6,7], so far no systematic evaluation of chronic skin changes in patients with an IRD induced by long-term systemic GC treatment has been performed to our knowledge.

We conducted this proof-of-concept study to investigate if HFUS employing an 18 MHz transducer of a high-end ultrasonography device commonly used in routine clinical care could demonstrate significant differences in dermal thickness comparing GC-treated patients with inflammatory rheumatic diseases (IRDs) and clinically evident skin atrophy to matched controls without IRDs and GC treatment.

## 2. Methods

### 2.1. Study Design

We performed a cross-sectional study evaluating dermal thickness by HFUS in a convenience cohort of GC-treated adult IRD patients with clinically evident skin atrophy and age- and sex-matched (1:1) controls.

Patients were recruited into the study at the time of hospital admission or outpatient clinic appointment during routine clinical care in a tertiary medical center specialized in the treatment of orthopedic and rheumatic diseases. They were included in the study if they had received a rheumatologist-established diagnosis of an IRD, had a cumulative duration of systemic GC treatment of at least 6 months, and had clinically evident skin atrophy, adjudicated by an experienced board-certified rheumatologist (one of the authors: B.E., M.F., or W.H.). For each recruited patient, an age- (equal decade of life) and sex-matched control was recruited among accompanying relatives or patients without the diagnosis of an IRD. Controls were excluded if they reported any current (in the last month) or prior prolonged (lifetime cumulative duration >2 weeks) topical or systemic GC treatment. Patients or controls with additional generalized dermatologic diseases other than mild untreated skin psoriasis, regardless of current or previous treatment with topical GC, or with any lesions in the six studied skin areas, were excluded.

All patients and controls provided written consent after receiving oral and written information about the study by one of the authors.

### 2.2. Demographic Data

Patients reported by supervised self-administered questionnaires if they had received any GCs in the previous week, in the previous month, or in the previous year and the cumulative duration and average daily prednisolone-equivalent dose of their GC treatment. The cumulative GC dose was approximated by multiplying the average daily dose by 365 and the cumulative GC duration in years and reported in grams. Additionally, patients reported the presence or absence of other common comorbidities, complications, or adverse effects of prolonged GC treatment: necessity for anticoagulation therapy, diabetes mellitus, arterial hypertension, hyperlipidemia, osteoporosis, cataract, and frequent or severe infections. Additional demographic data and the established diagnosis of an IRD was retrieved by chart review.

### 2.3. Clinical Examination

All patients were clinically examined on their entire body for the presence and severity of skin atrophy (none/mild/moderate/severe) and other common dermatologic adverse effects (none/mild/severe) of long-term GC treatment: purpura, striae rubrae (striae cutis distesae), facies lunata, and ‘Buffalo’ hump. We defined ‘mild’ skin atrophy for this study as localized but not generalized skin areas with parchment-like appearance, ‘moderate’ as skin with generalized parchment-like appearance, and ‘severe’ as generalized parchment-like skin with areas with a shiny surface.

### 2.4. High-Frequency Ultrasonography (HFUS)

All of the ultrasonography examinations were performed on a Logiq E9 device (GE Healthcare, Buckinghamshire, UK) with a linear hockey stick probe (8–18 MHz frequency). The technical parameters of the examination included a grayscale frequency of 18 MHz, an image depth of 1.3 cm, a focus position at 0.22 cm, and a gain of 45 dB, utilizing the presets for skin examinations provided by the manufacturer.

Separate HSUS B-mode images of the skin were acquired at 6 anatomical sites for each subject: the left and right back of the hand, longitudinally aligned over the metacarpal bone supporting the third digit; the left and right cubital area, longitudinally aligned 5 cm distal of the middle of the cubital skinfold; and the dorsal aspect of the left and right mid-food, longitudinally aligned over the metatarsal bone supporting the second digit.

**Figure 1:**
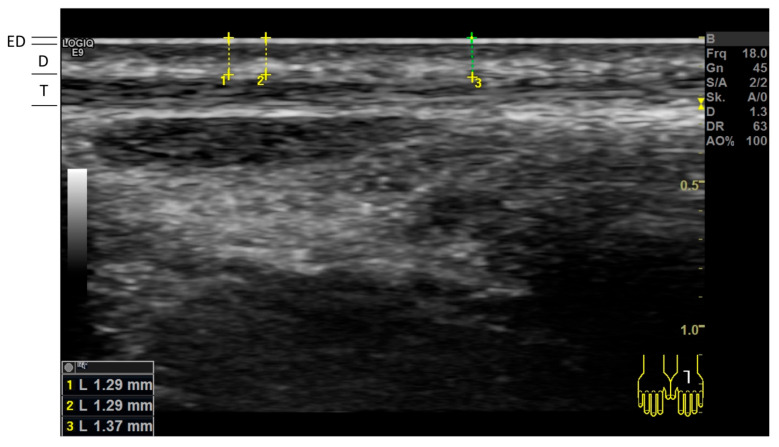
High-frequency ultrasonography of the dorsal left hand: epidermal (ED) layer, dermal (D) layer, and extensor tendon (T). Three measurements of the cutis thickness (ED and D layer combined) were performed for each image in areas with best discriminability of the border between the dermis and hypodermal structures.

To depict the exact cutis/subcutis border optimally and to enable exact perpendicular measurements to the surface of the skin, the linear probe was directly placed on the skin surface with the now-visible gel layer remaining between the probe and the skin, and a representative image for each anatomical region was acquired. On each image, three measurements of the cutis utilizing the device’s standard measuring tool (in mm, with two decimals) were performed at the image sections with the best discriminability of the border between the cutis and subcutis (Figure 1). For each anatomical site, the mean of the six measurements (3 left and 3 right) of the cutis (combined thickness of the epidermal and dermal layer) was calculated and used for all further analyses.

The skin of eight additional participants (not included in the main data set) was scanned and measured at the selected anatomical sites independently by three of the authors (B.E., A.H., and W.H.) to assess the interobserver reliability of the chosen HFUS skin measurement methodology. Intraclass correlation coefficients (ICCs), utilizing a two-way mixed model for the absolute agreement of single measurements, were calculated.

### 2.5. Caliper Measurement of Skinfolds

Additional to the clinical and HFUS evaluation, the skin in the six selected anatomical sites was also measured with a standardized mechanical Harpenden skinfold caliper utilizing an adapted methodology from a report by Dykes and coworkers [8]. For each site, two measurements were performed, and the mean of four measurements (2 left, 2 right) was calculated and used for further analyses. Due to the availability of the instrument, only some weeks after the initiation of the study, the first 14 enrolled subjects were not measured with the caliper.

### 2.6. Statistical Analyses

Due to the exploratory nature of our study, no formal sample size calculation was performed prior to enrolment of the convenience sample of 53 patients and 53 controls.

The primary endpoint of our study was to determine if the thickness of the cutis determined by HFUS and of skinfolds by the mechanical caliper differed significantly between the GC-treated IRD patients and controls. Additionally, secondary analyses of 4 subgroups (males < 70 years, males ≥ 70 years, females < 70 years, and females ≥ 70 years) were performed.

The continuous data were expressed as mean  ±  standard deviation (SD) and categorical data as number and percent. A comparison of continuous variables was performed by the Student’s *t*-test. For categorical variables, the chi-square or Fisher’s exact test were used. All statistical tests were 2-tailed, and a significance level (*p*) of 0.05 was used. The statistical tests were performed using SPSS for Windows version 26 (Chicago, IL, USA).

## 3. Results

First, the evaluation of eight separate patients for interobserver reliability of the employed imaging method by the three examiners revealed the ICCs for the mean cutis thickness at three anatomical sites of 0.749 (dorsal hand), 0.818 (cubital area), and 0.660 (dorsal mid-food), corresponding to a ‘good’, ‘excellent’, and ‘good’ interobserver reliability, respectively, according to the criteria set by D. Cicchetti [9]. 

Then, in the period from 1 April to 31 August 2018, 53 patients and 53 controls were recruited into the study. Demographical data, established IRD diagnoses, self-reported GC treatment, and non-dermatological adverse events associated with prolonged GC treatment are presented in Table 1. A majority of the 53 recruited IRD patients were female (63%), and 40% were older than 69 years. The most common IRDs were rheumatoid arthritis (RA, 49%), connective tissue diseases (11%), and polymyalgia rheumatica (11%). Enrolled patients with an IRD reported a mean duration of their IRD of 9 years and a mean duration of GC treatment of 10.4 years, corresponding to a mean calculated cumulative dose equivalent of approx. 30 g prednisone-equivalent. The controls had comparable age and sex distributions, but they reported significantly fewer pre-existing conditions known to be associated with long-term GC treatment (Table 1).

The comparison of patients and controls for clinical signs of GC-treatment-induced skin changes revealed significant differences for all signs other than striae rubrae (Table 2). Considering that clinical evident skin atrophy was an inclusion criterion, all 53 recruited patients presented with moderate (64%) or severe (36%) skin atrophy, while the majority of the sex- and age-matched controls had no (58%) or only mild (36%) skin atrophy in the clinical assessment. Skinfold thickness measured by the mechanical caliper at the back of hands [1.7 (±0.4) vs. 2.1 (±0.5) mm, *p* < 0.001] showed a significant difference between the two groups, but no significant differences could be delineated at the cubital [6.7 (±2.7) vs. 7.1 (±3.0) mm] or dorsal midfoot [3.6 (±3.7) vs. 4.1 (±3.4) mm] areas (Table 2).

**Figure 2:**
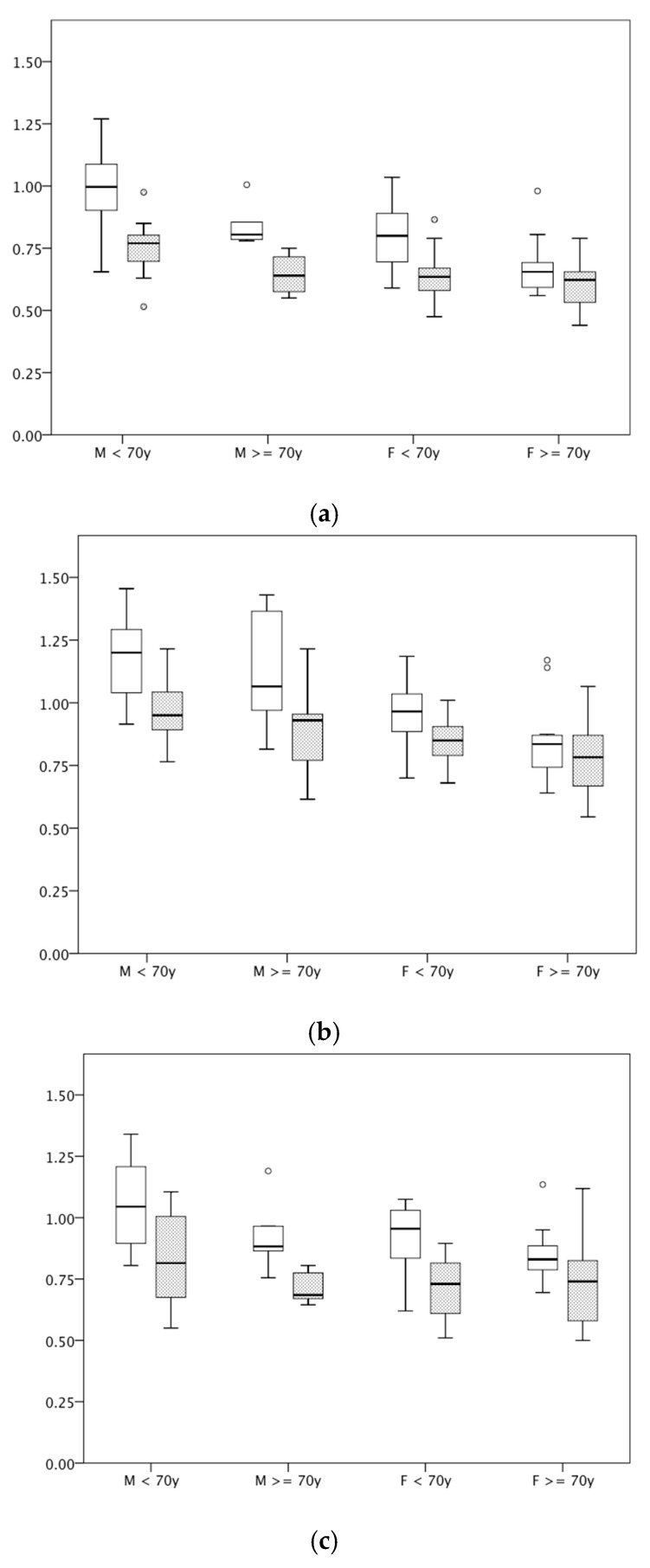
(**a**–**c**) Box plots of the cutis thickness (in mm) measured by high-frequency ultrasonography (HFUS) at the back of hand (**a**), cubital fossa (**b**), and dorsal midfoot (**c**) stratified by sex (M vs. F) and age (<70 vs. ≥70 years) for subjects without (white boxes) and patients with inflammatory rheumatic diseases and glucocorticoid use associated skin atrophy (gray boxes).

In comparison, all studied anatomical areas displayed statistically significant different cutaneous thickness in the evaluation by HFUS: back of hand 0.66 (±0.12) vs. 0.82 (±0.18) mm, *p* < 0.001; cubital area 0.86 (±0.15) vs. 1.00 (±0.21) mm, *p* < 0.001; and dorsal midfoot 0.76 (±0.16) vs. 0.94 (±0.18) mm, *p* < 0.001. Significant differences were also perceived in subgroup analyses for male or female patients aged below 70 years and for male patients above 70 years but not for female patients above 70 years (Table 3 and Figure 2a–c).

## 4. Discussion

In our study, we recruited 53 CS-treated patients with IRDs and clinically evident skin atrophy and 53 pair-matched controls without IRDs or GC treatment in this cross-sectional study to delineate the utility of HFUS to quantify skin atrophy. Our results demonstrated that patients had significantly lower cutaneous thickness measured by HFUS at all evaluated anatomical sites compared to controls. This significant difference was observed in three of the four evaluated subgroups, but not in women aged over 69 years.

Of course, sun exposure plays an important role in the occurrence of skin atrophy. Sun-exposed skin areas in particular are often affected [10]. Other skin lesions, such as purpura, are also associated with skin atrophy. The occurrence of purpura is also usually caused by skin atrophy as part of skin aging or after taking glucocorticoids or sun exposure and not by other medications, such as anticoagulants. In dermatology, the term dermatoporosis has become established to describe the skin changes that lead to the loss of skin function. The current EULAR guidelines for the treatment of rheumatoid arthritis stress the necessity and importance of a limited use of systemic glucocorticoids in long-term therapy. A rapid reduction and thereafter complete discontinuation of glucocorticoids is strongly recommended [11], given the well-documented side effects, including skin atrophy, which are associated with long-term use of glucocorticoids [3,12].

In particular, higher doses of glucocorticoids (e.g., prednisone-equivalent >7.5 mg/d) should be avoided to reduce side effects [12]. It should also be noted that inhaled glucocorticoids also have a certain systemic effect. For a more precise analysis of side effects, an international group of subspecialty physician experts have developed the Glucocorticoid Toxicity Index, with which glucocorticoid toxicity can be compared at different time points. In this study, the skin is included as a criterion that needs to be examined in that context [12]. However, even though skin atrophy is a known and well-studied side effect of both topical and systemic glucocorticoid use [13], there are currently no adequate standardized methods for recording and quantifying skin changes in routine clinical care.

In various studies, ultrasound has been evaluated as a possible tool for analyzing skin. In their study, Olsen et al. examined the skin thickness of 18 healthy volunteers using high-frequency ultrasound. They analyzed 22 different parts of the body to identify the differences between the various skin regions and the differences between men and women [14]. Women’s skin was thinner, which was also evident in our study. However, no statistically significant difference in skin thickness between the two groups could be found in women older than 70 years. Brincat et al. were able to show in their work that skin thickness decreases during menopause in women [15].

Several previous studies have investigated the use of ultrasound to analyze skin thickness after local topical glucocorticoid treatment. Korting et al. examined in their double-blind, placebo-controlled randomized study various topical glucocorticoids, which were used over 6 weeks in healthy volunteers. They were able to detect differences in skin atrophy between the different preparations, where prenicarbate caused less skin atrophy than betamethasone [16]. Also, Cossman et al. analyzed in their double-blind, placebo-controlled study the possibilities of using ultrasound examinations to measure skin thickness in healthy volunteers in the area of the volar part of both arms after topical glucocorticoid therapy for 4 weeks compared to placebo [17]. Here too, a decrease in dermal thickness was shown. Both studies were able to achieve results with a 20 MHz transducer, which was therefore comparable to the transducer used in our study [16,17].

In contrast, several studies in patients with systemic sclerosis have shown that ultrasound examinations can provide a good assessment of skin thickness both in the early stages of the disease and in contrast to healthy subjects [7,18,19]. Comparability to clinical assessment (mRSS) was also shown here [19].

Ultrasound examinations provide a fast and efficient assessment of the echogenicity and swelling of the skin, which allows drawing a conclusion about the tissue composition or the presence of inflammation. It also enables targeted therapy depending on the local tissue reaction. It also allows different disease entities and other complications to be differentiated from one another. However, a disadvantage is that the reproducibility of the examination is limited compared to other imaging techniques (MRI, CT, etc.), and the quality of the findings depends on the examiner’s experience. Sufficient training is therefore essential. The DERMUS Group, for example, recommends at least 300 ultrasound examinations per year in order to be considered sufficiently trained [20]. However, so far there are not enough studies on the use of high-frequency ultrasound in patients with long-term systemic glucocorticoid therapy, as in our study. Our study is therefore one of the first to show that HFUS can provide a good assessment of skin atrophy in patients with systemic glucocorticoids and that these patients experience a decrease in skin thickness compared to a control group without IRDs and glucocorticoid therapy in all examined locations.

The EULAR guidelines from 2010 already recommended that further studies should be carried out reflecting on the occurrence of skin changes during glucocorticoid therapy by determining epidermal and dermal skin thickness [21]. This recommendation shows the importance of our current study, especially considering that there are still no larger randomized studies on this subject.

A strength of our study is the use of a control group with comparable demographic characteristics as well as a standardized examination procedure, which also enabled a comparison between the different investigators. It should be noted that there are currently no internationally generalized and standardized guidelines for the use of ultrasound on the skin. So far, the generalizability of our findings is still limited due to the recruitment of patients treated at one tertiary referral center.

## 5. Conclusions

We conclude that our cross-sectional study revealed significantly lower cutaneous thickness measured by HFUS for GC-treated patients with IRDs and clinically evident chronic skin atrophy compared to matched controls without IRDs and GC treatment. Prospective studies evaluating patients with IRDs treated with prolonged courses of CS by HFUS should be performed to better define pharmacologic and demographic risk factors for the development of GC-induced cutaneous skin atrophy.

## Figures and Tables

**Table 1:** Demographics, rheumatic diagnosis, and pre-treatment with glucocorticoids for the 53 patients with and 53 age- and sex-matched subjects without inflammatory rheumatic diseases.

Subjects Studied	Patients with IRDs and GC > 6 mo.	Subjects Without IRDs and No Prior GC	*p*-Value *
	***n = 53***	***n = 53***	
***Demographics***			
Female sex, *n* (%)	33 (62)	32 (60)	0.842
Age in years, mean (±SD)	66.4 (±10.0)	65.9 (±11.3)	0.813
Age ≥ 70 years, *n* (%)	21 (40)	21 (40)	1.000
Body mass index in kg/m^2^, mean (±SD)	26.7 (±4.6)	27.3 (±5.3)	0.503
Duration since IRD diagnosis in years, median (range)	9 (0–47)	-	-
***Inflammatory rheumatic diseases (IRDs)***			
Rheumatoid arthritis	26 (49)	-	-
Gouty arthritis	2 (4)	-	-
Undifferentiated arthritis	2 (4)	-	-
Psoriatic arthritis	2 (4)	-	-
Spondyloarthritis	2 (4)	-	-
Connective tissue diseases	6 (11)	-	-
Polymyalgia rheumatica	6 (11)	-	-
Giant cell arteritis	5 (9)	-	-
Granulomatosis with polyangiitis	1 (2)	-	-
Polyarteritis nodosa	1 (2)	-	-
***Patient-reported treatment with glucocorticoids (GC)***			
Any GC in previous week, *n* (%)	52 (98)	-	-
Any GC in previous month, *n* (%)	52 (98)	-	-
Any GC in previous year, *n* (%)	52 (98)	-	-
Cumulative duration of GC treatment (years)			
mean (±SD)	10.4 (±10.0)	-	-
Average daily prednisolone-equivalent dose (mg)			
mean (±SD)	9.5 (±7.8)	-	-
Cumulative prednisolone dose (gram)			
mean (±SD)	30.5 (±38.5)	-	-
***Selected patient-reported pre-existing conditions***			
Anticoagulation therapy, *n* (%)	19 (36)	7 (13)	0.007
Diabetes mellitus, *n* (%)	9 (17)	4 (8)	0.139
Arterial hypertension, *n* (%)	40 (76)	21 (34)	<0.001
Hyperlipidemia, *n* (%)	20 (38)	9 (17)	0.017
Osteoporosis, *n* (%)	31 (59)	4 (8)	<0.001
Cataract, *n* (%)	16 (30)	4 (8)	0.003
More than 3 upper respiratory infections per year, *n* (%)	17 (32)	1 (2)	<0.001
Ever had an infection treated in the hospital, *n* (%)	15 (28)	2 (4)	0.001
Ever had an infection treated in intensive care, *n* (%)	8 (15)	0 (0)	0.006

* Statistical comparisons were performed utilizing Chi^2^− or Fischer’s exact test when appropriate. IRDs, inflammatory rheumatic diseases; GC, glucocorticoids; SD, standard deviation.

**Table 2:** Clinical signs, skinfold thickness measured by caliper, and cutaneous thickness measured by high-frequency ultrasonography (HFUS) for the 53 patients with and 53 age- and sex-matched subjects without inflammatory rheumatic diseases and prolonged glucocorticoid treatment.

Subjects Studied	Patients with IRD and GC > 6 mo.	Subjects Without IRD and No Prior GC	*p*-Value *
***Clinical signs***	***n = 53***	***n = 53***	
**Skin atrophy**			
None	0 (0)	31 (58)	<0.001
Mild	0 (0)	19 (36)	
Moderate	34 (64)	3 (6)	
Severe	19 (36)	0 (0)	
**Purpura**			
None	24 (45)	49 (92)	<0.001
Mild	17 (32)	4 (8)	
Severe	12 (23)	0 (0)	
**Striae rubrae**			
None	45 (85)	44 (83)	0.279
Mild	2 (4)	6 (11)	
Severe	6 (11)	3 (6)	
**Facies lunata**			
None	42 (79)	53 (100)	0.001
Mild	5 (10)	0 (0)	
Severe	6 (11)	0 (0)	
**Buffalo hump**			
None	41 (77)	51 (96)	0.010
Mild	8 (15)	2 (4)	
Severe	4 (8)	0 (0)	
***Skinfold thickness measured by caliper (mm)***	***n = 42 ^#^***	***n = 50 ^#^***	
Back of hand			
mean (±SD)	1.7 (±0.4)	2.1 (±0.5)	<0.001
Cubital fossa			
mean (±SD)	6.7 (±2.7)	7.1 (±3.0)	0.419
Dorsal midfoot			
mean (±SD)	3.6 (±3.7)	4.1 (±3.4)	0.501
***Cutis thickness measured by HFUS (mm)***	***n = 53***	***n = 53***	
Back of hand			
mean (±SD)	0.66 (±0.12)	0.82 (±0.18)	<0.001
Cubital fossa			
mean (±SD)	0.86 (±0.15)	1.00 (±0.21)	<0.001
Dorsal midfoot			
mean (±SD)	0.76 (±0.16)	0.94 (±0.18)	<0.001

Values are given as numbers of patients (% of column) if not stated otherwise. * Statistical comparisons were performed utilizing Chi^2^− or Fischer’s exact test when appropriate for categorical variables and Student’s *t*-test (continuous variables). ^#^ The first 14 enrolled patients did not receive clinical skin measurements due to lack of availability of the standardized caliper. IRD, inflammatory rheumatic diseases; GC, glucocorticoids; SD, standard deviation.

**Table 3:** Cutaneous thickness measured by high-resolution ultrasonography (HFUS) for the 53 patients with and 53 age- and sex-matched subjects without inflammatory rheumatic diseases and prolonged glucocorticoid treatment stratified by sex and age.

Cutaneous Thickness by HFUS (in mm)	Patients with IRD and GC > 6 mo.	Subjects Without IRD and No Prior GC	*p*-Value *
*Male, <70 years*	*n = 15*	*n = 15*	
Back of hand	0.75 (±0.11)	0.99 (±0.17)	<0.001
Cubital fossa	0.97 (±0.11)	1.18 (±0.16)	<0.001
Dorsal midfoot	0.84 (±0.19)	1.05 (±0.18)	0.004
*Male, ≥70 years*	*n = 5*	*n = 6*	
Back of hand mean	0.65 (±0.09)	0.84 (±0.17)	0.005
Cubital fossa	0.90 (±0.22)	1.12 (±0.24)	0.148
Dorsal midfoot	0.72 (±0.07)	0.92 (±0.15)	0.018
*Female, <70 years*	*n = 17*	*n = 17*	
Back of hand	0.64 (±0.10)	0.80 (±0.13)	0.001
Cubital fossa	0.84 (±0.09)	0.94 (±0.13)	0.016
Dorsal midfoot	0.72 (±0.12)	0.93 (±0.13)	<0.001
*Female, ≥70 years*	*n = 16*	*n = 15*	
Back of hand mean	0.61 (±0.11)	0.67 (±0.11)	0.131
Cubital fossa	0.78 (±0.15)	0.84 (±0.15)	0.309
Dorsal midfoot	0.74 (±0.18)	0.85 (±0.15)	0.057

Values are given as mean (±SD). * Statistical comparisons were performed utilizing Student’s *t*-test. IRD, inflammatory rheumatic diseases; GC, glucocorticoids; SD, standard deviation.

## Data Availability

The data sets used and/or analyzed during the current study are available from the corresponding author on reasonable request.

## Abbreviations

GCs	glucocorticoids
HFUS	high-frequency ultrasonography
IRDs	inflammatory rheumatic diseases
ICC	intraclass correlation coefficients

## References

[1] Oray M., Abu Samra K., Ebrahimiadib N., Meese H., Foster C.S. (2016). Long-term side effects of glucocorticoids. Expert. Opin. Drug Saf..

[2] Curtis J.R., Westfall A.O., Allison J., Bijlsma J.W., Freeman A., George V., Kovac S.H., Spettell C.M., Saag K.G. (2006). Population-based assessment of adverse events associated with long-term glucocorticoid use. Arthritis Rheum..

[3] Amann J., Wessels A.-M., Breitenfeldt F., Huscher D., Bijlsma J.W.J., Jacobs J.W.G., Buttgereit F. (2017). Quantifying cutaneous adverse effects of systemic glucocorticoids in patients with rheumatoid arthritis: A cross-sectional cohort study. Clin. Exp. Rheumatol..

[4] Polańska A., Dańczak-Pazdrowska A., Jałowska M., Żaba R., Adamski Z. (2017). Current applications of high-frequency ultrasonography in dermatology. Postep. Dermatol. Alergol..

[5] Barnes L., Kaya G., Rollason V. (2015). Topical corticosteroid-induced skin atrophy: A comprehensive review. Drug Saf..

[6] Hesselstrand R., Carlestam J., Wildt M., Sandqvist G., Andréasson K. (2015). High frequency ultrasound of skin involvement in systemic sclerosis—A follow-up study. Arthritis Res. Ther..

[7] Naredo E., Pascau J., Damjanov N., Lepri G., Gordaliza P.M., Janta I., Ovalles-Bonilla J.G., López-Longo F.J., Matucci-Cerinic M. (2020). Performance of ultra-high-frequency ultrasound in the evaluation of skin involvement in systemic sclerosis: A preliminary report. Rheumatology.

[8] Dykes P.J., Marks R. (1977). Measurement of skin thickness: A comparison of two in vivo techniques with a conventional histometric method. J. Investig. Dermatol..

[9] Cicchetti D.V. (1994). Multiple comparison methods: Establishing guidelines for their valid application in neuropsychological research. J. Clin. Exp. Neuropsychol..

[10] Battie C., Jitsukawa S., Bernerd F., Del Bino S., Marionnet C., Verschoore M. (2014). New insights in photoaging, UVA induced damage and skin types. Exp. Dermatol..

[11] Smolen J.S., Landewé R.B.M., Bergstra S.A., Kerschbaumer A., Sepriano A., Aletaha D., Caporali R., Edwards C.J., Hyrich K.L., Pope J.E. (2023). EULAR recommendations for the management of rheumatoid arthritis with synthetic and biological disease-modifying antirheumatic drugs: 2022 update. Ann. Rheum. Dis..

[12] Huscher D., Thiele K., Gromnica-Ihle E., Hein G., Demary W., Dreher R., Zink A., Buttgereit F. (2009). Dose-related patterns of glucocorticoid-induced side effects. Ann. Rheum. Dis..

[13] Stone J.H., McDowell P.J., Jayne D.R., Merkel P.A., Robson J., Patel N.J., Zhang Y., Yue H., Bekker P., Heaney L.G. (2022). The glucocorticoid toxicity index: Measuring change in glucocorticoid toxicity over time. Semin. Arthritis Rheum..

[14] Olsen L.O., Takiwaki H., Serup J. (1995). High-frequency ultrasound characterization of normal skin. Skin thickness and echographic density of 22 anatomical sites. Skin. Res. Technol..

[15] Brincat M., Kabalan S., Studd J., Moniz C., de Trafford J., Montgomery J. (1987). A study of the decrease of skin collagen content, skin thickness, and bone mass in the postmenopausal woman. Obstet. Gynecol..

[16] Korting H.C., Unholzer A., Schäfer-Korting M., Tausch I., Gassmueller J., Nietsch K.-H. (2002). Different skin thinning potential of equipotent medium-strength glucocorticoids. Skin. Pharmacol. Appl. Skin. Physiol..

[17] Cossmann M., Welzel J. (2006). Evaluation of the atrophogenic potential of different glucocorticoids using optical coherence tomography, 20-MHz ultrasound and profilometry; a double-blind, placebo-controlled trial. Br. J. Dermatol..

[18] Lepri G., Hughes M., Allanore Y., Denton C.P., Furst D.E., Wang Y., Santiago T., Galetti I., Del Galdo F., Khanna D. (2023). The role of skin ultrasound in systemic sclerosis: Looking below the surface to understand disease evolution. Lancet Rheumatol..

[19] Flower V.A., Barratt S.L., Hart D.J., Mackenzie A.B., Shipley J.A., Ward S.G., Pauling J.D. (2021). High-frequency Ultrasound Assessment of Systemic Sclerosis Skin Involvement: Intraobserver Repeatability and Relationship with Clinician Assessment and Dermal Collagen Content. J. Rheumatol..

[20] Beiu C., Popa L.G., Bălăceanu-Gurău B., Iliescu C.A., Racoviță A., Popescu M.N., Mihai M.M. (2023). Personalization of Minimally-Invasive Aesthetic Procedures with the Use of Ultrasound Compared to Alternative Imaging Modalities. Diagnostics.

[21] van der Goes M.C., Jacobs J.W.G., Boers M., Andrews T., Blom-Bakkers M.A.M., Buttgereit F., Caeyers N., Cutolo M., Silva J.A.P.D., Guillevin L. (2010). Monitoring adverse events of low-dose glucocorticoid therapy: EULAR recommendations for clinical trials and daily practice. Ann. Rheum. Dis..

